# Central Positional Nystagmus: A Systematic Literature Review

**DOI:** 10.3389/fneur.2017.00141

**Published:** 2017-04-20

**Authors:** Nora K. Macdonald, Diego Kaski, Yougan Saman, Amal Al-Shaikh Sulaiman, Amal Anwer, Doris-Eva Bamiou

**Affiliations:** ^1^Neuro-otology Department, National Hospital for Neurology and Neurosurgery, London, UK; ^2^UCL Ear Institute, London, UK; ^3^Department of Otolaryngology and Head and Neck Surgery, King Fahd Hospital of University, University of Dammam, Al-Khobar, Saudi Arabia

**Keywords:** nystagmus, vertigo, central positional nystagmus, central positional vertigo, positional nystagmus, positional vertigo

## Abstract

**Objective:**

To provide a systematic review of the clinical and radiological features of lesion-induced central positional nystagmus (CPN) and identify salient characteristics that differentiate central from peripheral positional nystagmus (PN).

**Methods:**

Systematic literature search according to the preferred reporting items for systematic reviews and meta-analysis.

**Results:**

A total of 82 patients from 28 studies met the participants intervention, comparison, outcomes, and study designs criteria for inclusion. An atypical direction of nystagmus for the stimulated canal was reported in 97.5% patients during Dix–Hallpike (D–H) and 54.5% upon supine roll testing. Five types of CPNs were identified during positional testing: positional horizontal nystagmus (pHN) (36.8%), positional downbeating nystagmus (pDBN) (29.2%), positional torsional nystagmus (pTN) (2.1%), positional upbeating nystagmus (pUBN) (2.1%), and a combination of the four profiles (29.9%). CPN was paroxysmal (<60 s) in 85% patients on straight head hanging (SHH), 63.9% on D–H, and 37.5% on supine roll, and had a latency <3 s upon positioning in 94.7% patients in which it was reported. Concurrent vertigo was reportedly present in 63.4% patients and 48.8% demonstrated other neurological signs. Radiologically, in 74.4%, there was mention of cerebellar involvement, isolated brainstem involvement in 8.5%, and 14.6% involved the fourth ventricle.

**Conclusion:**

Currently, there is a lack of robust data on the clinical and radiological characteristics of CPN highlighting the need for better phenotyping of CPN to help differentiate this entity from peripheral causes of PN. With increased awareness of CPN, particularly in the acute setting, we may see a change in the estimated prevalence of CPN and improved clinical markers to promptly identify the frequently sinister underlying causes.

## Introduction

Diagnosis of a central positional syndrome can be challenging. Currently, it is based on the presence of deviations to the diagnostic criteria for benign paroxysmal positional vertigo (BPPV) (1), and the clinical profile has otherwise received little attention. Atypical or infrequent variants of BPPV (2), interchangeable use of terminology, and absence of widely accepted definitions for central positional syndromes all add to the diagnostic challenges. Positional nystagmus (PN) is defined as the nystagmus generated by a change in head position with respect to gravity (3). It has been classified according to site of lesion (peripheral versus central), temporal nystagmus characteristics (paroxysmal versus persistent) (4), or a combination of temporal and directional nystagmus characteristics (type 1 = persistent, direction changing, type 2 = persistent, direction-fixed, and type 3 = transitory) (5). The majority of studies, however, use additional nystagmus characteristics of latency, duration, and fatigue (Table 1), and response to repositioning maneuvers, in order to differentiate central positional nystagmus (CPN) from the peripheral PN attributed to BPPV and its variants (3). Rather confusingly, the terms CPN and central *positional vertigo* are also used interchangeably. Since CPN may occur both with and without vertigo, and its features are the hallmark for diagnosis of central positional syndromes versus BPPV, the present paper will adopt the term CPN (with or without vertigo) as the clinical entity of interest.

**Table 1:** **Typical clinical features of peripheral BPPV and central PPV [Adapted from Ref. (1, 3, 6)]**.

Features	BPPV	Central PPV
Latency following precipitating positioning manoeuver	1–5 s (shorter in h-BPPV depending on acceleration of head turn and cupulolithiasis)	0–5 s
Duration of nystagmus	5–60 s (longer in cupulolithiasis)	5 to >60 s
Direction of nystagmus	During stimulation in the plane of the affected canal; torsional/vertical for p-BPPV and a-BPPV; horizontal for h-BPPV	Pure vertical; pure torsional, not attributable to the stimulated canal plane
Fatiguability	Typical for pc-BPPV and a-BPPV, rare in h-BPPV	Possible
Course of nystagmus and vertigo in an attack	Crescendo–decrescendo typical, not common in h-BPPV	Crescendo–decrescendo possible
Vertigo	Typical	Typical, with exceptions
Nausea and vomiting	Rare on single precipitating maneuvers (associated with intense nystagmus, not uncommon after several maneuvers)	Frequent on single precipitating maneuvers (not necessarily) associated with strong nystagmus intensity
Natural course of the condition	Spontaneous recovery within several weeks in 70–80%	Spontaneous recovery rare
Associated neurological signs and symptoms	None	Often cerebellar and brainstem oculomotor signs
Brain imaging	Normal	Lesions of the dorsal vermis and/or dorsolateral to the fourth ventricle
Repositioning therapy	Positional nystagmus disappears after appropriate positional therapy	Refractory to repositioning therapy

*BPPV, benign paroxysmal positioning vertigo/nystagmus; PPV, paroxysmal positioning vertigo/nystagmus; a, anterior; h, horizontal; p, posterior canal*.

No studies have rigorously assessed the prevalence of CPN; however, retrospective studies from neuro-otology (7) and falls clinics’ clinical setups (8) report that 11–12% of PNs are central and thus not rare within these clinical settings. In view of the potentially sinister causes of CPN, it is important that clinicians are able to diagnose this clinical entity effectively and promptly. Clinical data on CPN are currently sparse so the clinically preferred diagnostic approach is to “rule in a peripheral cause of PN rather than to rule out CPN.” It is, however, a well-known clinical notion that vertical PN, lack of latency, and long duration of nystagmus may indicate CPN, but to date this has not been evaluated in a systematic way.

The objective of this study was to review the clinical features and radiological findings of lesion induced CPN to (i) identify parameters fundamental in the assessment of CPN and (ii) identify salient characteristics that differentiate central from peripheral PN.

## Methods

### Requirement for Review

This preliminary search included a search of the Database of Abstracts of Reviews of Effects, which is produced by the NIHR Centre for Reviews and Dissemination (CRD) (and contains details of all Cochrane Reviews, Protocols for Cochrane Reviews, and other publications based on Cochrane Reviews), in addition to the National Institute for Health and Clinical excellence (NICE) database and the Campbell Library of Systematic reviews.

The CRD Database yielded 12 results for “positional vertigo” or “central positional vertigo” in “any field,” none of which discussed PN/vertigo due to a central, rather than peripheral, origin. A search on the Cochrane Library produced six articles for “positional vertigo” in “all text” and two articles for “central positional vertigo” in “all fields,” all of which related to BPPV, rather than central positional vertigo. A “central positional vertigo” search on the NICE database revealed three results on BPPV. The Campbell Collaboration Library of systematic reviews gave 0 HITs for “positional vertigo” and “central positional vertigo” in “all text,” “keywords,” and “title.”

### Systematic Search Strategy and Study Selection

A review protocol was formulated based on guidelines for systematic reviews in health care (9, 10).

Study inclusion criteria were formulated using the participants, intervention, comparison, outcomes and study designs (PICOS) strategy (Table 2). To avoid author bias, advisors and second authors were assigned to review the outcomes and provide independent input at appropriate stages in the process. Any disagreement was resolved by consensus or third party adjudication.

**Table 2:** **Participants, intervention, comparison, outcomes, and study designs criteria for inclusion**.

Patient population	Adult (+18 years)
	Presenting with PN and/or vertigo confirmed as central in origin
	Any setting
Intervention	Intervention must include positional testing as a means of observing the PN
Comparison	Not applicable
Outcome	The clinical presentation of the PN must be reported in terms of at least one of the following characteristics: direction, provoking position, duration, and latency
Study design	Published work from all study designs
Date	No limitation
Language	English, French, German
Publication type	Must be peer-reviewed

### Inclusion Criteria

#### Participants

We included studies of adults (>18 years) complaining of, or presenting with, PN and/or vertigo caused by confirmed central nervous system (CNS) pathology. Studies were included if the CNS pathology was confirmed either on imaging (MRI or CT), autopsy, following surgical intervention or other examinations, such as cerebrospinal fluid analysis. Non-humans studies were excluded.

#### Interventions

We included studies that used positional maneuvers as a means of observing CPN across hospital, university, or research settings.

#### Outcomes Measures

##### Primary Outcomes

We included studies that reported descriptive or quantitative data relating to the clinical presentation of CPN upon positional testing including, but not limited to, the direction of nystagmus, provoking position, latency of onset, duration, co-existing symptoms, and fatigability. Studies that failed to comment on any one of these characteristics of the PN were excluded.

##### Secondary Outcomes

Additional study outcomes include the clinical examination, atiology/imaging findings, symptoms, and management plan.

#### Study Design

The initial search was for randomized controlled trials and controlled trials. However, when information from controlled trials was not available, cohort studies were eligible for inclusion. The intention was to exclude case series and case reports from the review due to the high potential for bias in these study designs. However, if there proved to be a lack of cohort studies available, published work from all study designs were accepted, including case series and case reports.

#### Language

Studies were accepted for inclusion in the review if they were English, French, or German, with the exception of translations of publications.

### Study Identification

#### Database Search

A sensitive systematic protocol for database searching was adopted. Each topic was defined individually for the database using the “or” element. In this review, the individual topics were (central/non-benign) and (PN/vertigo). “And” was then used to connect the topic defining “or” searches to locate papers directly relating to PN or vertigo that was central or non-benign (rather than BPPV).

To ensure sensitivity of the search, “exp” (Explode) was used to search for any papers that have been assigned to the database subject heading “positional,” “vertigo,” “nystagmus,” or “pathologic,” and for any papers assigned below “positional vertigo,” “nystagmus,” or “pathologic” in the hierarchy. The “$” was applied to request the database to search for multiple possible endings to the word “position.” Positional was included in the search by means of using the “adj” (adjacent) operator. “adj3” was used to retrieve any instances of the word position/positional/positions/positioning, occurring within (three) words of vertigo, nystagmus, or vertiginous in the title or abstract of a paper.

Searches of electronic journals were conducted using the Cochrane Ear, Nose, and Throat, MEDLINE (PUBMED), the Cochrane database, and EMBASE. Google scholar and Yahoo Internet searches were also employed to identify any additional relevant material. There was no date restriction applied to the search. Searches were updated on a monthly basis between March 2014 and February 2017.

#### Searching Other Resources

The reference lists of identified studies were scanned for further studies.

### Screening

**Figure 1:**
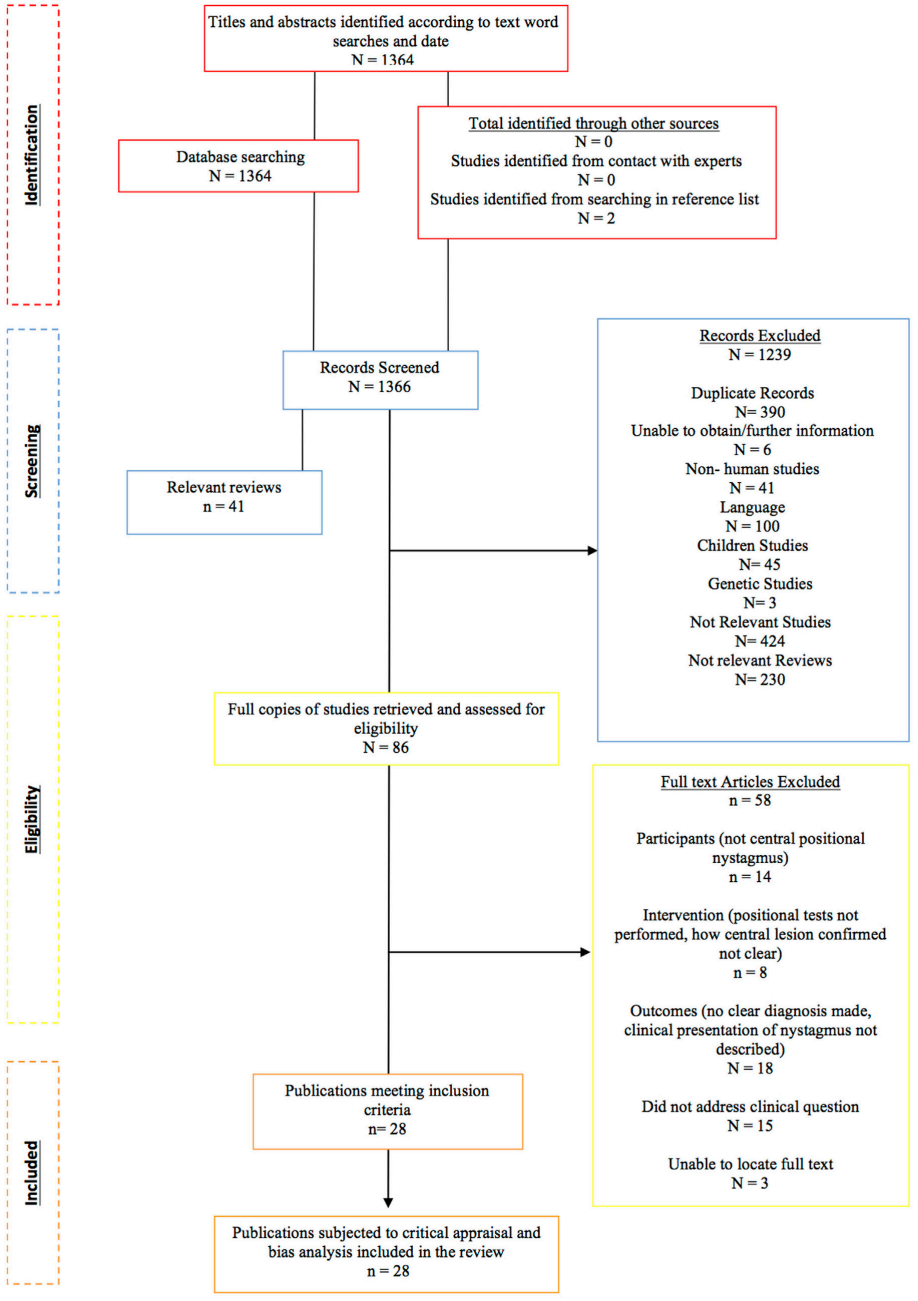
**Flow chart of the study identification, eligibility, and inclusion process**.

The process of study identification and selection (recommended by preferred reporting items for systematic reviews and meta-analysis) is demonstrated in Figure 1.

The database searches returned a total of 1,364 articles. No further articles were identified through the additional journal searches including reference lists and contact with experts. After the removal of duplicates (*n* = 390) and reviews (*n* = 41), a total of 933 potentially relevant studies remained. The abstracts of these articles were assessed by the primary author relative to the study identification protocol of which 847 failed to meet the inclusion criteria. Studies were deemed “non-relevant” if they did not report the clinical presentation or diagnosis of central positional vertigo/nystagmus. Studies that highlighted atypical BPPV rather than CPV were sub-grouped for the purposes of further discussion, not to be included in the primary review. The titles and abstracts of the excluded articles were independently verified by two second authors.

Where insufficient detail was available in the abstract to make a decision, the paper proceeded to the second stage of assessment, where the full text would be retrieved. A total of 86 studies either met the PICOS criteria for inclusion based on the abstract or contained insufficient information from which to make a judgment, and progressed to the second stage of screening where the full-text was obtained.

#### Eligibility

Four authors (Nora K. Macdonald, Doris-Eva Bamiou, Yougan Saman, and Amal Al-Shaikh Sulaiman) independently reviewed the full texts of the remaining 86 studies for eligibility to ascertain the final studies to be included in the review. Any disagreement was resolved by consensus or third party adjudication. A total of 28 studies were eligible for inclusion in the systematic review. The reasons for rejecting the remaining articles are demonstrated in e-Bibliography. The accepted records were grouped by study design type, of which there were two: case studies and case series.

#### Potential Sources of Bias

The study selection process of this review did not identify any controlled studies relevant to the diagnosis of central positional vertigo/nystagmus. For this reason, this review included case studies and case series for consideration of their evidence. The authors recognize that such study designs, which are retrospective in nature, are inherently susceptible to the risk of bias. The case series did not typically recruit consecutive series of individuals from multiple centers, and this introduced a risk of selection bias. The studies were also at risk of detection of reporting/observer bias in which the clinical presentation of the CPN reported was not standardized. Furthermore, none of these case studies or case series that were considered relevant to the current review referred to results in a comparison group.

Despite the methodological difficulties associated with case studies/series, the authors viewed this lack of high quality evidence as justification for the review. For this reason, the review proceeded despite the inclusion of only case studies and all their associated scientific weakness.

### Data Extraction

#### Study Design

This included the design of the study (case series or case study) as well as the number of participants and the study setting.

#### Participants

This included demographic details of the subject/s with reference to relevant medical history and history of the positional vertigo.

#### Characteristic of PN

The characteristics of nystagmus upon positional testing were extracted from each patient. This description was divided into the classical features of PN, i.e., the direction of nystagmus, provoking position, duration of nystagmus, latency of onset, fatigability, and the presence of concurrent symptoms such as subjective vertigo, nausea, and/or vomiting.

#### Site of Lesion

The site of lesion was extracted from each subject. This information was in the form of imaging results, autopsy reports, or reports from surgical investigations.

#### Associated Signs and Symptoms

Associated neurological signs or symptoms, or the absence there-of, were noted for each subject. This information referred to any central symptom such as gait disturbances, abnormal oculomotor function, and atypical symptoms such as unexplained weight loss, as well as reference to normal or abnormal observations upon clinical examination and testing.

#### Management

Although not considered a primary outcome of this review, the respective management of each subject was extracted. This information, when present, was considered relevant in order to investigate any potential failure to respond to repositioning maneuvers, which may contribute to the diagnosis of a central, rather than peripheral, origin. In addition, reports from any surgical intervention may stand as evidence of the site of lesion.

## Results

### Study Characteristics

A total of 28 (out of 1,364) identified studies met the PICOS criteria for review inclusion (see Figure 1). The characteristics of the study set are described in terms of the PICOS criteria.

#### Participants

Participant samples in the included papers involved individuals with CPN, i.e., had atypical positional or positioning nystagmus attributed to a confirmed central lesion. Subjects within each of the studies’ sample who did not have confirmed CPN were excluded. This included 88 participants in the study by Bertholon et al. (7) and 33 participants in Maire and Duvoisin (11) with peripheral PN.

Included studies ranged in (relevant) sample sizes from single case subjects (12–25) up to 14 subjects (26–29).

In total, there were 82 participants (age range 20–81 years) from 14 countries, with the majority (84%) from University settings. It was not possible to establish the mean age and the male/female ratio of the included participants due to insufficient demographic data.

Seventy-two (87.8%) study participants had central pathologies confirmed on either MRI (4, 6, 12, 13, 15–20, 22, 24, 30, 31) or CT (14, 21, 22, 31–34) imaging, and two (2.4%) upon autopsy examination (32, 34). In the remaining eight (9.8%), CNS pathologies were confirmed on imaging, but nine participants for whom the diagnostic means were not specified were excluded from the review.

#### Intervention

Positional testing was used to provoke the CPN in all participants (see Tables S1 and S2 in Supplementary Material). In 13 (15.9%) cases, CPN was triggered by all three Dix–Hallpike (D–H), supine roll, and straight head hanging (SHH) maneuvers (4). In five (6.1%) cases, CPN was reported on both D–H and SHH (4, 16), while in one case, the CPN was triggered by D–H and supine with head to the left (18).

In 33 (40.2%) cases, D–H alone was used to trigger CPN (7, 11, 12, 14, 17, 19, 25, 27, 31, 34). CPN was triggered only upon lying supine with either ear down in 26 (31.7%) cases (6, 7, 13, 15, 20–24, 26, 28, 32–35) and only upon SHH in 3 (3.7%) (6, 31, 33). In one case, CPN was triggered by SHH and horizontal head rotation with either ear down (26).

A repeat of the positional test (to observe a fatigue effect) was reported in 28 (34.1%) subjects (6, 12, 13, 15, 17, 19, 22, 31, 34).

Additional evaluation often included a clinical or objective examination of oculomotor (85.4%) (4, 6, 7, 11–19, 22, 24–26, 28–30, 32, 34, 35) and gait (41.5%) function (6, 14–17, 19, 22, 26, 27, 30, 31, 34).

#### Outcomes Measures

The means of assessing the observed nystagmus upon positional testing was poorly reported. PN was quantified in 51 (62.1%) participants, 35 (42.7%) with VNG (4, 15, 20–26, 35), 15 (18.3%) with electronystagmography (11, 14, 18, 33), and 1 (1.2%) using three-dimensional scleral induction coil (6). It is uncertain how the nystagmus was recorded in the remaining 31 participants (37.8%), presumably from direct observation during clinical examination (6, 7, 12, 13, 16, 17, 19, 30–34).

#### Study Designs

The study set included 11 case series (6, 7, 11, 26, 30–36) and 17 case reports (12–25, 27–29). This review did not identify any controlled studies relevant to the diagnosis of central positional vertigo/nystagmus.

### Nystagmus Profile

Table S1 in Supplementary Material describes the CPN characteristics in each patient provoked only by D–H, while Table S2 in Supplementary Material describes that observed upon further positional testing.

#### Direction of CPN

The direction of nystagmus and provoking positional test were sufficiently reported in 70 (85.4%) patients only, and atypical direction of the nystagmus, on the basis of that predicted for the canal stimulated during positional testing, was a prominent feature.

##### Dix–Hallpike

Of the 52 patients with CPN provoked by D–H, 40 (76.9%) cases reported the direction. CPN was purely vertical in 19 of the 40 (47.5%) patients (7, 14, 16, 19, 27, 30), purely horizontal in 5 (12.5%) (7, 18, 34), purely torsional (rotatory) in 2 (5%) (12, 34), and was a combination involving a downbeating component in 13 (32.5%) (7, 17, 30). Of the 40 (48.8%) participants with positive D–H, only 1 demonstrated nystagmus in the direction considered “typical” of BPPV (31).

The purely vertical nystagmus upon D–H was pure downbeating in 17 (42.5%) (7, 14, 16, 27, 30), pure upbeating in 1 (2.5%) (19), and upbeating followed by downbeating in 1 (2.5%) (7). Due to a lack of consensus in terminology, it was impossible to interpret the direction of torsional nystagmus unless specified, but nystagmus beating away from the lowermost ear (apogeotropic) was reported by Choi et al. (4) in all nine of the patients who were presented with torsional nystagmus upon D–H testing (4).

Of the 40 patients who had a positive D–H and reported the direction of nystagmus, 24 (60%) were reported to be positive to both left and right ear down positions (7, 16, 17, 27, 30, 34), which changed the direction of the nystagmus in 15 (62.5%) (7, 17, 30, 34).

##### Roll Test

Positional nystagmus upon horizontal plane (roll) head movements while supine was reported in 41 (50%) cases (6, 7, 13, 15, 18, 20, 22, 24, 28, 29, 32, 33), at least 18 (43.9%) of which were atypical in terms of direction (6, 7, 13, 15, 18, 32, 33).

Rolling the head to one side while supine resulted in horizontal nystagmus with a rotatory component in four (9.8%) (13, 32, 33), purely positional torsional (rotatory) nystagmus (pTN) in one (2.4%) (6), positional upbeating nystagmus (pUBN) in one (2.4%) (6), and horizontal with an upbeating component in two (4.9%) (28, 29). Although 33 patients (80.5%) presented with exclusively positional horizontal nystagmus (pHN) upon supine roll, this was not present to both sides in 9 (23.1%) (7, 18, 32) and was direction-reversing while the position was maintained in 1 (2.6%) (15), therefore, considered atypical of lateral canal BPPV. Lee et al. (35) did not report whether the pHN was present to both left and right head positions, or just one, in his patients. Of the eight, Lee et al report six (75%) with ageotropic and two (25%) with geotropic pHN.

All 15 of the remaining pHN in this study set, which reversed when the head was moved to the opposite side (and, therefore, could be considered typical of lateral canal BPPV), were apogeotropic (4, 20–24, 26).

##### Straight Head Hanging

Positional nystagmus upon SHH was reported in 22 (26.7%) patients (6, 16, 26, 31, 33), with 17 (77.3%) exclusively positional downbeating (pDBN) (4, 6, 16, 31), 4 (18.2%) downbeating with rotatory component (4), and 1 (4.5%) horizontal with rotatory component (33).

##### Diagnostic Positional Test Overall Findings

Overall, across all participants and maneuvers, the direction of CPN was reported on 144 occasions. Five types of CPNs were identified during positional testing: pHN on 53 (36.8%) occasions (7, 15, 18, 20–24, 26, 32, 34, 35), pDBN was reported on 42 (29.2%) (6, 7, 14, 16, 26, 27, 30, 31), purely pTN in 3 (2.1%) (6, 12, 34), and exclusively pUBN in 3 (2.1%) (6, 19). A combination of the profiles was demonstrated in the remaining 43 (29.9%) (7, 13, 16, 17, 28–30, 32, 33).

A pDBN (component) was provoked in 34 (41.5%) patients. It was triggered by both angular deflection of the head upon D–H testing or SHH maneuvers in 17 (20.7%) patients (7, 14, 16, 17, 30), by D–H only in 16 (19.5%) patients (7, 14, 17, 25, 27, 30), and SHH position only in 3 (3.7%) cases (6, 16, 26, 31).

Direction-reversing PN while the provoking position was maintained was reported in 3 (3.7%) of the 82 participants (7, 15, 16).

#### Duration of CPN

The duration of CPN upon D–H was reported in 36 (43.9%) patients, the majority of which was paroxysmal (*n* = 21, 58.3%), lasting less than 16 s in 19 (52.8%) (4, 14, 16, 19, 27, 34) and “transient” in 2 (5.6%) (31, 34). In the remaining 15 (41.7%), the CPN upon D–H lasted at least a minute (11, 17), or was referred to as “persistent” (12, 18, 30).

Of the 20 subjects in which duration was reported upon SHH, 17 (85%) demonstrated transient CPN (of <16 s) (4, 6, 16). CPN persisted for over 1 min upon SHH in the remaining 3 (15%) subjects (4, 33).

In contrast, of the 24 subjects who reported the duration of CPN upon supine roll testing, the majority (*n* = 15, 62.5%) was persistent, lasting at least a minute (4, 6, 13, 18, 22, 24, 32, 33), while the remaining 9 (37.5%) was short-lived (less than 1 min) (4, 6, 15, 20).

Of the 35 patients with pDBN, 25 (71.4%) reported its duration. pDBN was short-lived (<17 s) in 21 (60%) cases (6, 14, 16, 27) and persisted for at least 1 min in only 4 (11.4%) subjects.

In total, across all positional tests, CPN persisted for at least 1 min in 33 out of 81 (40.7%) in occasions it was reported.

#### Latency of CPN

Latency of onset of PN upon assuming the provoking position was reported in 38 (46%) patients. Nystagmus “without latency” upon positioning was observed in 30 out of 38 (78.9%) patients (4, 6, 15–17, 19, 21, 22, 25, 30, 32, 34). Six (15.8%) reported CPN with a latency lasting either a “few” seconds, <2 s, or with a “short” latency upon positioning (6, 13, 14, 30, 31, 34). The remaining two cases (5.3%) reported CPN with an onset >3 s (3–5 and >10 s) upon positioning (6, 18).

pDBN developed after a short latency period (<2 s) in all six (100%) cases in which this feature was reported (6, 14, 16, 30, 31).

#### Habituation, Fatigue, and Natural Course

The nystagmus response upon repeated testing was reported in 28 (34.1%) participants. The CPN fatigued with repeated maneuvers only in 5 (17.9%) of these cases (12, 13, 31, 34), but did not fatigue in the remaining 23 (82.1%) (6, 15–20, 22, 34).

Nystagmus with no remission was demonstrated by Imai and colleagues (20) who reported a patient’s pHN beating away from the lowermost ear on left and right supine that continued without remission for 1,600 days. No patient (*n* = 16) (13, 19, 22, 24–26, 35) responded to repositioning maneuvers.

#### Fixation

The effect of fixation on CPN was only reported in four studies. Maire and Duvoisin (11) found that, in a sample of 43 patients with static PN, the predictive value of the ocular fixation test was 94% (*n* = 35) for peripheral lesions and a 100% (*n* = 8) for central disorders, with the latter being associated with reduced optic fixation index. Buttner et al. (1, 6) reported poor vestibular ocular reflex (VOR) suppression (6), while Williams et al. (25) reported normal VOR suppression (25). Furthermore, Barber (14) reported pDBN, which was indeed enhanced with fixation, and Cobb and Friedman (18) reported nystagmus, which disappeared when fixation was removed.

### Vertigo and Nausea and/or Vomiting

Positional vertigo was referred to in 55 (67.1%). Of the 55, subjective vertigo accompanied CPN in 52 (94.5%) (4, 6, 14–16, 26–30, 34–36). Associated nausea was commented on 18 patients, 15 (83.3%) of which reported vertigo with intense nausea/vomiting (6, 12, 15, 27, 30–34).

### Neurological Signs and Symptoms

Almost half (*n* = 40, 48.8%) of the patients demonstrated at least one central symptom or abnormality (6, 7, 11, 12, 14–17, 22, 27–34).

Neurological symptoms other than vertigo were reported on 56 occasions. These included gait unsteadiness (*n* = 23) (6, 15–18, 22, 23, 25, 27, 30, 33) with falls (*n* = 3) (7, 31), headache (*n* = 7) (31, 32, 34, 35), motor weakness (*n* = 4) (6, 16, 32), clumsiness of extremities (*n* = 3) (18, 31), unexpected weight loss (*n* = 2) (14, 32), diplopia (*n* = 2) (20, 32), asymmetrical hearing loss (*n* = 4) (28, 29, 32, 35), facial numbness (*n* = 2) (6, 35), slurred speech (*n* = 1) (16), dysphagia (*n* = 1) (35), oscillopsia (*n* = 2) (28, 32), visual blurring (*n* = 1) (12), and loss of taste (*n* = 1) (32).

Cerebellar and other central oculomotor signs were also prevalent in CPN. Within our data set, of the 70 (85.4%) participants in which oculomotor function was reported, 41 (58.6%) demonstrated either gaze-evoked nystagmus, abnormal saccades, and/or broken pursuit (4, 6, 7, 11, 12, 17, 25–27, 34). Eighteen participants had unspecified ataxia (4, 6, 14, 23, 27, 31, 34).

Normal eye movements were reported in six participants (11, 13, 14, 18, 26) and a completely “normal neurological evaluation” in 31 (6, 13, 18–20, 22–24, 29, 31, 35).

### Radiological Features

Of the 82 review participants, 61 underwent MRI brain imaging (74.4%), 11 underwent CT brain imaging (13.4%), and in 2, participants’ lesions were determined on the basis of autopsy findings (2.4%). In the remaining eight participants (9.8%), imaging was undertaken, but the modalities were not specified.

**Figure 2:**
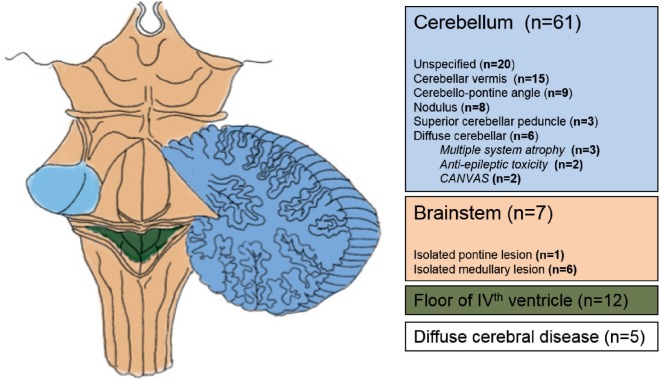
**Anatomical areas affected in patients with central positional nystagmus based on imaging and autopsy data**.

Figure 2 illustrates and summarizes the reported lesion locations with their relative frequency across the review studies. In 61 participants (74.4%), there was cerebellar involvement (4, 6, 7, 11–17, 21–23, 26–35). Seven participants (8.5%) had isolated brainstem lesions (6, 19, 20, 22, 23, 35), and 12 participants (14.6%) had lesions involving the fourth ventricle (11, 13, 26, 33, 34). In five participants (6.1%), lesions were “diffuse” (16, 18) or unspecified (7).

Nausea/vomiting was reported in 15 patients (32.9%) (6, 12, 15, 20, 27–29, 31, 32, 34). Of these 15, 9 (60%) had space occupying lesions (6, 12, 15, 27, 31–34). Three (20%) of these 15 patients with nausea/vomiting had inflammatory lesions in the brachium conjunctivum of the superior cerebellar peduncle (30) and 3 (20%) patients had posterior inferior cerebellar artery infarcts (6, 29).

## Discussion

Central positional nystagmus arises due to disruption of brainstem or cerebellar vestibular networks, but making a confident differential diagnosis from peripheral PN remains a clinical challenge. We have reviewed the available literature on CPN using a systematic search in an attempt to better define CPN and identify differentiating features from peripheral PN. Perhaps unexpectedly, given the number of reports of CPN in the literature, the absence of robust and systematic clinical data provided in the majority of these reports highlights the ongoing need to define the topographical basis of CPN and reliably identify the salient characteristics that help distinguish peripheral from central PN. One major strength of this, the largest review of CPN to date, is the extensive clinical and oculographic data collated using consistent and clinically relevant categories. Analysis of data with such broad methodologies and varied clinical reporting raises a number of shortfalls: case studies/series, which are retrospective in nature, are susceptible to the risk of detection or outcome reporting bias. The clinical presentation of the CPN reported was not standardized using detailed oculography and clinical examination reports lacked important clinical outcomes. Associated symptoms such as vertigo, nausea, and other neurological symptoms were subject to recall and positive reporter bias. Furthermore, none of the case studies/series herein referred to results in a comparison group. Single case studies often lacked important clinical data. From a radiological perspective, imaging data were mostly described within the data set in the absence of the MRI images.

We now discuss the salient findings.

### Nystagmus Characteristics of CPN

Table 3 summarizes the frequency of the nystagmus characteristics and associated neurological features for central (versus peripheral) PN from our data set.

**Table 3:** **Frequency of the typical indicators of central positional nystagmus**.

Criterion	Frequency (%)	Instances reported
The direction is not attributable to the stimulated canal plane	72.2	97
Pure vertical nystagmus	31.3	144
Pure torsional nystagmus	2.1	144
Direction-reversing nystagmus while the position is maintained	3.7	82
Enhancement with fixation or reduced ocular fixation index	91.7	12
It persists for at least 1 min or as long as the precipitating head position is maintained	40.7	81
Commences with no latency or within 3 s of assuming the provoking positon	94.7	38
Does not fatigue with repetitive positioning	82.1	28
Additional oculomotor abnormalities	58.6	70
Additional brainstem or cerebellar symptoms and/or abnormalities	48.8	82
PN does not resolve with repeated repositioning maneuvers	100	16
Prominent nausea or vomiting on positioning	83.3	18
Prominent vertigo on positioning	94.5	55

#### Direction

It is noteworthy that only 2 (2.4%) patients within the data set reported the presence of spontaneous nystagmus (12, 25). This minimized the potential influence of the well-known phenomenon that a discrete spontaneous downbeat nystagmus, which may not be visible under Frenzel glasses, becomes only evident by positioning the patient.

While it is proposed that a central origin has to be assumed for pure upbeat, downbeat, and torsional nystagmus (1), we found that in about a one-third of cases (29.9%), CPN was a mixture of horizontal, torsional, and vertical components in variable combinations depending on the positioning maneuver performed. Furthermore, the direction of CPN is not always “atypical” for the plane of the canal being stimulated, since downbeat nystagmus was the most prominent direction during SHH (in 77.3%) while pHN was most prominent upon supine head turning (in 80.5%). Theoretically, a symmetrical bilateral p-BPPV could lead to paroxysmal upbeat nystagmus during coactivation of both posterior semicircular canals in the SHH position. For HC-BPPV, the nystagmus that occurs when the left ear or right ear is in the down position can be directed to the undermost (geotropic) or uppermost (apogeotropic) ear (1). Thus, this feature (geotropic or apogeotropic) cannot be used to differentiate a peripheral or central origin.

Only one patient had a positive D–H with the direction of nystagmus characteristic of BPPV (31) but failed to respond to repositioning treatments, and later developed ataxia. Cho and colleagues (26) presented three patients who, except for apogeotropic PN during supine roll tests, had normal neurological examinations. After an initial diagnosis of BPPV, canalith repositioning maneuvers were applied repeatedly but without success, and the patients were later found to have central lesions. These cases highlight the importance of considering central causes of PN when presumed BPPV is refractory to treatment.

#### Direction Changing versus Direction Reversing

Direction-changing nystagmus is described in the literature as nystagmus whose fast phase direction changes (e.g., from right-beating to left-beating) when the position of the head changes (37). Direction-changing pHN on roll test strongly favors the diagnosis of HC-BPPV (7). However, direction-changing nystagmus was noted in 62.5% of CPN participants on D–H (4, 7, 16, 17, 25, 30, 34) and should alert to the presence of CPN during the D–H.

Direction reversing is a different phenomenon, developing spontaneously in two successive phases after the patient reaches a particular position (and without further head movement). It has been suggested that the first phase represents the pathological nystagmus caused by a central vestibular disorder (vestibulocerebellar dysfunction), while the secondary nystagmus represents the adaptive mechanism to nullify the original pathological nystagmus (15). Direction reversing was only reported in three (3.9%) CPN participants and may have a high positive predictive value for central pathology, but relatively low negative predictive value.

#### Duration and Latency

Our data set demonstrates a high prevalence of central paroxysmal positional nystagmus (CPPN), which predominantly decays in under 30 s on D–H and SHH. However, CPN triggered by supine head roll typically lasted at least 1 min, mimicking the observations of cupulolithiatic HC-BPPV (1). Choi et al. (4) proposed that the temporal patterns of nystagmus intensity distinguished the two disorders (4).

In our data set, latency proved to be a move reliable indicator of CPN with 94.7% commencing within 3 s (78.9% starting “with no latency”) of assuming the provoking position. Despite the diagnostic strength of this parameter, it was only reported in less than half (46%) of patients within this data set.

#### Ocular Fixation

The loss of visual suppression of nystagmus with optic fixation indicates a lesion at the flocculonodular lobe of the cerebellum (38, 39). The effect of visual fixation upon nystagmus was only documented in two patients of the papers reviewed here, with patients with posterior fossa lesions demonstrating a failure of fixation suppression (16).

### Associated Features of CPN

Central positional vomiting refers to the presence of vomiting triggered by a positioning maneuver and was reported in 15 (out of 18 participants on whom the symptom was commented upon). A positive reporting bias, therefore, could not be excluded. Central positional vomiting may occur in the absence of nystagmus or vertigo (40, 41). This review found that vomiting almost always occurred in the presence of vertigo in a variety of space occupying lesions. The cause of vomiting may be due to lesion-related pressure effects on the area postrema in the caudal aspect of the floor for the fourth ventricle (33), or related to affectation of cerebellar and brainstem pathways involved in the integration of vestibular and non-vestibular afferents relating to body position in space (42).

In several papers, especially those from otolaryngology settings, it is not clear to what extent a thorough neurological examination had been conducted and whether neurological symptoms/signs were under-reported. Bertholon et al. (7) found that CPN was not an isolated oculomotor finding in 10 out of 12 (83%) patients with confirmed CNS pathologies (7). In these cases, CPN was associated with gaze-evoked nystagmus and/or abnormal smooth pursuit. A majority of patients (60.9%) with CPN had associated neurological symptoms, most commonly gait unsteadiness, and neurological signs, including gaze-evoked nystagmus and ataxia.

### Clinico-Radiological Association and Pathophysiology

All patients with CPN had lesions that involved the cerebellum and brainstem (Figure 2). From a practical clinical perspective, the presence of CPN is thus highly predictive of lesions in the posterior fossa, involving a communicating network between the vestibular apparatus (otolith organs and semicircular canals), brainstem vestibular nuclei, and midline cerebellar structures within the vermis. Choi et al. (4) formulated an elegant hypothesis of how CPPN may be generated on the basis of nodular and uvular disinhibition of irregular afferent signals converging on the vestibular nucleus, but more detailed tractography studies into the communication between these subcortical vestibular regions may be needed to extend this hypothesis to all types of CPN (4). The neuronal mechanisms underlying persistent CPN are unknown but may involve the velocity-storage mechanism, which prolongs the afferent vestibular signal from the semicircular canals and may also be involved in the segregation of tilt and translation (43, 44).

Given the heterogeneity of the data set reported, we propose that there may be distinct clinico-radiological or clinico-pathological CPN syndromes. Studies assessing positional oculographic data and associated neurological features in distinct clinical syndromes (e.g., leukoaraiosis, multiple system atrophy) or in patients with discrete brainstem/cerebellar lesions are required to take the proposition forward.

It is important to note that BPPV is very common and may co-exist with brain structural/functional pathology, e.g., superimposed BPPV on DBN in Bertholon et al. (16), or in SCA 6 as demonstrated by Yu-Wai-Man et al. (45). With this in mind, the presence of features atypical for BPPV (Table 3) should warrant further investigation, even if BPPV has been diagnosed. Similarly, positional vertigo may be the only precursor symptom of a CNS disorder, e.g., in SCA6, before the onset of ataxia/other neurological abnormalities at follow-up.

## Future Research

In a landmark study, Choi et al. (4) systematically analyzed CPN in all positions using oculography and provided state-of-the-art imaging data. However, the oculographic features investigated were limited to the nystagmus direction, nystagmus duration, and etiology of CPPN. There is, therefore, a pressing need for more detailed clinical phenotyping of patients with CPN syndromes, with a view to developing classification systems to aid diagnosis of potentially sinister central disorders. This will require the systematic use of oculography to explore the characteristics of PN, an appreciation of accompanying neurological features, of vertigo, vomiting, and brainstem signs in particular, detailed neurological examination, and brain magnetic resonance imaging.

A formulation of the mechanism underlying CPN will allow the translation of descriptive neurological investigation into pathophysiological mechanisms that can inform therapeutics, but this requires a prior understanding of the clinical syndromes.

## Summary

This review sheds light on CPN as a much underdiagnosed and neglected topic in neurology. With an increasing awareness of this entity, a better understanding of the underlying mechanism of central vertigo, and ever-improving diagnostic tools, we may see a change in the estimated prevalence of central positional vertigo, and improved clinical markers to distinguish these from more benign peripheral causes.

## Author Contributions

NM contributed to design the systematic review protocol; collected, analyzed, and interpreted the data; and drafted the manuscript, tables, and figures. DK analyzed and interpreted the data; provided fundamental radiological interpretation of the results; and drafted the manuscript, tables, and figures. D-EB led the design of the protocol; contributed to the stratification of papers and extraction of data; and provided critical revisions to the manuscript. YS and AS contributed to the stratification of papers and extraction of data. AA contributed to the development of the systematic review protocol. All contributing authors read and approved the final manuscript.

## Conflict of Interest Statement

The authors declare that the research was conducted in the absence of any commercial or financial relationships that could be construed as a potential conflict of interest.
