# Supplementary material for: Central Positional Nystagmus: A Systematic Literature Review
Source: Front Neurol. 2017 Apr 20;8:141. doi: 10.3389/fneur.2017.00141 (PMC5397512; doi:10.3389/fneur.2017.00141)
Supplement: Supplementary file 1 [file Table_1.DOCX]

Supplementary Material

Central Positional Nystagmus: A Systematic Literature Review

**NK Macdonald, MSc^1*^, D Kaski, PhD^2*^, Y Saman, PhD^1^, A Al-Shaikh Sulaiman, PhD^2 ,^ A Anwer, MSc^2^ , DE Bamiou^1,2^ PhD**

*** Correspondence:** Nora Macdonald, [nora.macdonald.10@ucl.ac.uk](mailto:nora.macdonald.10@ucl.ac.uk),

# Supplementary e-Table 1. Characteristic of CPN upon Dix-Hallpike testing according to Etiology

| No. | Reference | Etiology | Location of Lesion | Direction of CPN | | Duration (s) | Latency (s) | Vertigo | Nausea/ Vomiting | Fatiguability |
| --- | --- | --- | --- | --- | --- | --- | --- | --- | --- | --- |
|  |  |  |  | R D-H | L D-H |  |  |  |  |  |
| 1 | Anagnostou et al (2008) | MS | SCP (L) | CC |  | +++ | - | + | + | + |
| 2 | Anagnostou et al (2006) | MS | SCP (R) | DB & LB | DB | +++ | < 2 | + | + |  |
| 3 |  | MS | SCP (R) | DB | DB | +++ |  | + | + |  |
| 4 | Bertholon (2006) | MSA |  | DB | |  |  |  |  |  |
| 5 |  | Atrophy | Diffuse | DB | |  |  |  |  |  |
| 6 |  | Atrophy (Degenerative disorders) | Diffuse | DB | |  |  |  |  |  |
| 7 |  | Atrophy | Cbll | DB | |  |  |  |  |  |
| 8 |  | MS |  | DB | |  |  |  |  |  |
| 9 |  | Infarct | Brain (not specified) | DB | |  |  |  |  |  |
| 10 |  | Carbamazepine toxicity |  | DB | |  |  |  |  |  |
| 11 |  | Atrophy | Cbll | UB / DB | UB /DB |  |  |  |  |  |
| 12 |  | Infarct | Cbll | Ageotropic Horizontal | |  |  |  |  |  |
| 13 |  | MSA |  | DB / RB | DB / LB |  |  |  |  |  |
| 14 | Beyon J, et al (2000) | Cholesteatoma | CPA (L) | RB & DB & C | LB & DB & CC | > 60 | - |  |  | - |
| 15 | Habek et. Al (2010) | Infarct | Pons | UB | - | 15 | - | + | - | - |
| 16 | Kattah et al (1984) | Cystic mass | Cbll vermis | DB | DB | 10 |  | + | + | + |
| 17 | Maire and Duvoisin (1999) | Tumour | IV ventricle |  |  | > 60 |  |  |  |  |
| 18 |  | Tumour | IV ventricle |  |  | > 60 |  |  |  |  |
| 19 |  | Infarction | CPA |  |  | > 60 |  |  |  |  |
| 20 |  | Infarction | CPA |  |  | > 60 |  |  |  |  |
| 21 |  | Haemorrhagic concussion | CPA |  |  | > 60 |  |  |  |  |
| 22 |  | MS | CPA |  |  | > 60 |  |  |  |  |
| 23 |  | Ptroclival Mengioma | CPA |  |  | > 60 |  |  |  |  |
| 24 |  | Atrophy | Cbll |  |  | > 60 |  |  |  |  |
| 25 |  | Atrophy | Cbll |  |  | > 60 |  |  |  |  |
| 26 |  | Epidermoid cyst | Cbll vermis |  |  | > 60 |  |  |  |  |
| 27 | Shoman and Longdridge (2007) | Tumour/Astocytoma | Cbll vermis | - | “positive” | transient |  | + | + | + |
| 28 | Watson et al (1981) | Tumour | IV ventricle | RB | LB |  | - | + | + | - |
| 29 |  | Tumour | IV ventricle | CC | - | 5-10 | 3-5 | + | + | + |
| 30 |  | Astrocytoma | Cbll (R) |  | RB | 5 | - | - | - | - |
| 31 |  | Unknown obstruction | IV ventricle | RB | - | transient | “few” | + | - | + |
| 32 | Williams et al (2013) | Gangliocytoma | Cbll (R) | DB & C | RB & C |  | - |  |  | - |
| Abbreviations: + = present; - = nil/absent; +++ = persistent/sustained; / = followed by; & = accompanied by; C = clockwise; CC = counterclockwise; Cbll = cerebellum; Cbll vermis = cerebellar vermis; CPA = cerebellopontine angle; DB = down-beating; D-H = Dix Hallpike; L = left; LB= left beating; MS = multiple sclerosis; PF= posterior fossa; R = right; RB= right beating; SCP = superior cerebellar peduncle; UB = up-beating.  = Not Reported | | | | | | | | | | |
