# Supplementary material for: Central Positional Nystagmus: A Systematic Literature Review
Source: Front Neurol. 2017 Apr 20;8:141. doi: 10.3389/fneur.2017.00141 (PMC5397512; doi:10.3389/fneur.2017.00141)
Supplement: Supplementary file 2 [file Table_2.DOCX]

Supplementary Material

Central Positional Nystagmus: A Systematic Literature Review

**NK Macdonald, MSc^1*^, D Kaski, PhD^2*^, Y Saman, PhD^1^, A Al-Shaikh Sulaiman, PhD^2 ,^ A Anwer, MSc^2^ , DE Bamiou^1,2^ PhD**

*** Correspondence:** Nora Macdonald, [nora.macdonald.10@ucl.ac.uk](mailto:nora.macdonald.10@ucl.ac.uk),

# Supplementary e-Table 2. Characteristic of CPN testing according to Etiology

|  |  | | | DIX-HALLPIKE | | | | | | | | | | | | | HEAD MOVEMENTS IN HORIZONTAL PLANE WHILE SUPINE | | | | | | | STRAIGHT HEAD HANGING | | | | | |
| --- | --- | --- | --- | --- | --- | --- | --- | --- | --- | --- | --- | --- | --- | --- | --- | --- | --- | --- | --- | --- | --- | --- | --- | --- | --- | --- | --- | --- | --- |
|  | Reference | Etiology | Location of Lesion | Direction | | Duration (s) | | Latency (s) | | Vertigo | | Nausea/ Vomiting | | Fatigability  y | | | Direction | | Duration (s) | Latency (s) | Vertigo | Nausea/ Vomiting | Fatigability | Direction | Duration (s) | Latency (s) | Vertigo | Nausea/ Vomiting | Fatigability |
|  |  |  |  | R | L | R | L | R | L | R | L | R | L | R | L | | R | L |  |  |  |  |  |  |  |  |  |  |  |
| 1 | Arai and Terakawa  (2005) | Infarct | Dorsolateral to the 4^th^ ventricle |  | |  | |  | |  | |  | |  | | | - | LB +T | 60 | “Few” | + |  | + |  |  |  |  |  |  |
| 2 | Barber (1984) | Lesion (Unspecified) | Cbll vermis | DB | | 5-6 | | 1-2 | | + | |  | |  | | |  |  |  |  |  |  |  |  |  |  | + |  |  |
| 3 | Bassani, Della Tore (2011) | Tumour | Cbll |  | |  | |  | |  | |  | |  | | | DR Geotropic/ageotropic | | 10 – 20 | - | + | + |  | - | - | - | + | + | - |
| 4 | Bertholon et al (2003) | Atrophy | CPA | DB /UB | DB | 10 | Short-lived | - | - / 3-5 | + | - |  | | - / + | - | |  |  |  |  |  |  |  | DB | “short-lived” | - | - |  | - |
| 5 | Bertholon (2006) | Infarct | Cbll |  |  |  |  |  |  |  |  |  | |  |  | | - | RB |  |  |  |  |  |  |  |  |  |  |  |
| 6 | Büttner (1998) | Infarct | Medulla |  | |  | |  | |  | |  | |  | | | - | T | 25-30 | - | + | + | - |  |  |  |  |  | - |
| 7 |  | Infarct | Medulla |  | |  | |  | |  | |  | |  | | | UB | UB | 60 | - | + | + |  |  |  |  |  |  |  |
| 8 |  | Lesions | Cbll |  | |  | |  | |  | |  | |  | | |  |  |  |  |  |  |  | DB | 10-15 | < 2 | + | + | - |
| 9 | Cho et al (2017) | Tumour | Cbll |  |  |  | |  | |  | |  | |  | |  | LB | RB |  |  | + |  |  | DB |  |  | + |  |  |
| 10 |  | Tumour | Cbll |  |  |  | |  | |  | |  | |  | |  | LB | RB |  |  | + |  |  |  |  |  |  |  |  |
| 11 |  | Tumour | Cbll |  |  |  | |  | |  | |  | |  | |  | LB | RB |  |  | + |  |  |  |  |  |  |  |  |
| 12 | Choi et al (2015) | Infarction | Cbll | DB + CC | DB + C | 10-12 | | - | | + | |  | |  | | LB | RB | >60 |  | + |  |  | DB + CC | 7-16 | - | + |  |  |  |
| 13 |  | Tumour | Cbll | DB + CC | DB + C | 10-11 | | - | | + | |  | |  | | LB | - | >60 |  | + |  |  | DB + CC | 7-16 | - | + |  |  |  |
| 14 |  | Infarction | Cbll | DB | DB | 10-12 | | - | | + | |  | |  | | LB | - | >60 |  | + |  |  | DB | 7-16 | - | + |  |  |  |
| 15 |  | Infarction | Cbll | DB + CC | DB + C | 14-16 | | - | | + | |  | |  | | LB | - | <60 |  | + |  |  | DB + CC | 7-16 | - | + |  |  |  |
| 16 |  | Infarction | Cbll | DB | DB | 7-8 | | - | | + | |  | |  | | LB | RB | >60 |  | + |  |  | DB | 7-16 | - | + |  |  |  |
| 17 |  | Infarction | Cbll | DB + CC | DB + C | 10-11 | | - | | + | |  | |  | | LB | - | >60 |  | + |  |  | DB | 7-16 | - | + |  |  |  |
| 18 |  | Infarction | Cbll | DB | DB | 11-12 | | - | | + | |  | |  | | LB | RB | <60 |  | + |  |  | DB | 7-16 | - | + |  |  |  |
| 19 |  | Infarction | Cbll | DB + CC | DB + C | 10-11 | | - | | + | |  | |  | | - | - | - | - | + |  |  | DB | 7-16 | - | + |  |  |  |
| 20 |  | Hemorrhage | Cbll vermis | DB | DB | 12-13 | | - | | + | |  | |  | | LB | RB | <60 |  | + |  |  | DB | 7-16 | - | + |  |  |  |
| 21 |  | Tumour | Cbll vermis | DB + CC | DB + C | 7-8 | | - | | + | |  | |  | | LB | RB | <60 |  | + |  |  | DB | 7-16 | - | + |  |  |  |
| 22 |  | Tumour | Cbll vermis | DB + CC | DB + C | 7-8 | | - | | + | |  | |  | | LB | - | >60 |  | + |  |  | DB | 7-16 | - | + |  |  |  |
| 23 |  | Infarction | Cbll vermis | DB / LB* | DB/RB* | 10-11/ >60* | | - | | + | |  | |  | | - | - | - |  | + |  |  | DB | 7-16 | - | + |  |  |  |
| 24 |  | Tumour | Cbll | DB + CC | DB + C | 14-15 | | - | | + | |  | |  | | LB | RB | <60 |  | + |  |  | DB + CC | 7-16 | - | + |  |  |  |
| 25 |  | Infarction | Cbll | DB + CC | DB + C |  | | - | | + | |  | |  | | LB | - | <60 |  | + |  |  | DB | >60 | - | + |  |  |  |
| 26 |  | MSA |  | DB/LB* | DB/RB* | 11-12/ >60* | | - | | + | |  | |  | | - | - | - |  | + |  |  | DB | 7-16 | - | + |  |  |  |
| 27 |  | CANVAS |  | DB | DB | 14-15 | | - | | + | |  | |  | | LB | RB | >60 |  | + |  |  | DB | 7-16 | - | + |  |  |  |
| 28 |  | AED Toxicity |  | DB | DB |  | | - | | + | |  | |  | | - | - | - |  | - |  |  | DB | >60 | - | + |  |  |  |
| 29 | Cobb and Friedman (2006) | Atrophy/ ischaemic disease | Fronto-parietal and peripventricualr | LB | - | +++ | | 137 | | - | |  | | - | |  | - | RB | +++ | 10-25 | - |  | - |  |  |  |  |  |  |
| 30 | Goto, N. et al (1983) | Metastasis | Cbll |  | |  | |  | |  | |  | |  | | | LB | - | +++ | - | + |  |  |  |  |  |  |  |  |
| 31 |  | Lymphoma | CPA |  | |  | |  | |  | |  | |  | | | LB +T | |  |  | + | + |  |  |  |  |  |  |  |
| 32 | Imai, T. et al (2010) | Infarct | Medulla |  | |  | |  | |  | |  | |  | | | LB | RB | Transient |  | + |  | - |  |  |  |  |  |  |
| 33 | JohkuraJohkura (2007)Johkura Johkura | Haemorrhage | Cbll |  | |  | |  | |  | |  | |  | | | LB | RB |  | - |  |  |  |  |  |  |  |  |  |
| 34 | Kasarkas, A (1981) | Multiple metastases | IV ventricle |  | |  | |  | |  | |  | |  | | |  |  |  |  |  |  |  | RB + T | > 120 |  | + | + |  |
| 35 |  | Lesion | Cbll & IV ventricle |  | |  | |  | |  | |  | |  | | | LB + T | RB + T | >120 |  | + | + |  |  |  |  |  |  |  |
| 36 |  | Ependymoma | IV ventricle |  | |  | |  | |  | |  | |  | | | LB + T | RB + T | 120 |  | + | + |  |  |  |  |  |  |  |
| 37 | Kim, H. et al (2012) | Infarct | Nodulus |  | |  | |  | |  | |  | |  | | | LB | RB | +++ | - | - |  | - |  |  |  |  |  |  |
| 38 | Lee et al (2014) | hemangioblastoma | Cbll |  | |  | |  | |  | |  | |  | | | LB | RB |  |  | + |  | - |  |  |  |  |  |  |
| 39 |  | Infarct | PICA +AICA |  | |  | |  | |  | |  | |  | | | LB | RB |  |  | + |  | - |  |  |  |  |  |  |
| 40 |  | infarct | nodular |  | |  | |  | |  | |  | |  | | | LB | RB |  |  | + |  | - |  |  |  |  |  |  |
| 41 |  | tumour | cbll |  | |  | |  | |  | |  | |  | | | LB | RB |  |  | + |  | - |  |  |  |  |  |  |
| 42 |  | tumour | Bilateral Nodular |  | |  | |  | |  | |  | |  | | | LB | RB |  |  | + |  | - |  |  |  |  |  |  |
| 43 |  | infarct | Medulla |  | |  | |  | |  | |  | |  | | | LB | RB |  |  | + |  | - |  |  |  |  |  |  |
| 44 |  | tumour | Cbll |  | |  | |  | |  | |  | |  | | | RB | LB |  |  | + |  | - |  |  |  |  |  |  |
| 45 |  | infarct | medulla |  | |  | |  | |  | |  | |  | | | RB | LB |  |  | + |  | - |  |  |  |  |  |  |
| 46 | Nam et al (2009) | infarct | Bilateral nodular |  | |  | |  | |  | |  | |  | | | LB | RB |  |  |  |  |  |  |  |  |  |  |  |
| 47 | Shaikh (2014) | Infarct | Cbll vermis |  | |  | |  | |  | |  | |  | | | RB & UB | - |  |  |  |  |  |  |  |  |  |  |  |
| 48 | Shaikh et al (2014) | Ischaemia | Cbll vermis |  | |  | |  | |  | |  | |  | | | - | RB & UB |  |  |  |  |  |  |  |  |  |  |  |
| 49 | Shoman and Longdridge (2007) | Lesion | Cbll vermis | - | - | - | - | - | - | - | - | - | - |  | | |  |  |  |  |  |  |  | DB |  | short |  |  |  |
| 50 | Taylor et al (2012) | VS | CPA |  | |  | |  | |  | |  | |  | | | LB | RB | 120 |  |  |  |  |  |  |  |  |  |  |
| Abbreviations: + = present; - = nil/absent; +++ = persistent/sustained; / = followed by; & = accompanied by; AED = antiepileptic drug; BC = Brachium conjunctivum; CANVAS = Cerebellar ataxia-neuropathy-vestibular areflexia syndrome; C = clockwise; CC= counterclockwise; Cbll = cerebellum; CPA = cerebellopontine angle; DB= down-beating; DR = direction reversing; L = left; LB = left beating; MS = multiple sclerosis; MSA = multiple system atrophy; R = right; RB = right beating; SCP = superior cerebellar peduncle; T= torsional (unspecified direction); UB = Up-beating; VS = vestibular schwannoma.  = Not Reported  *patient reportedly had persistent ageotropic CPN, but due to insufficient information it is uncertain in what position this was present | | | | | | | | | | | | | | | | | | | | | | | | | | | | | |
