# Supplementary material for: Central Positional Nystagmus: A Systematic Literature Review
Source: Front Neurol. 2017 Apr 20;8:141. doi: 10.3389/fneur.2017.00141 (PMC5397512; doi:10.3389/fneur.2017.00141)
Supplement: Supplementary file 3 [file Data_Sheet_1.DOCX]

Supplementary Material

Central Positional Nystagmus: A Systematic Literature Review

**NK Macdonald, MSc^1*^, D Kaski, PhD^2*^, Y Saman, PhD^1^, A Al-Shaikh Sulaiman, PhD^2 ,^ A Anwer, MSc^2^ , DE Bamiou^1,2^ PhD**

*** Correspondence:** Nora Macdonald, [nora.macdonald.10@ucl.ac.uk](mailto:nora.macdonald.10@ucl.ac.uk),

# Supplementary Data

## e-Bibliography. Bibliography of excluded studies in Eligibility stage

### Reason for exclusion: did not address clinical question

1. Beyea JA, Parnes LS. Purely vertical upbeat nystagmus in bilateral posterior canal benign paroxysmal positional vertigo: a case report. Laryngoscope 2010;120:208-209.
2. Cambi J, Astore S, Mandala M, Trabalzini F, Nuti D. Natural course of positional down-beating nystagmus of peripheral origin. Journal of Neurology 2013;260:1489-1496.
3. Dumas G, Charachon R, Lavieille JP. Benign positioning vertigo (BPV) and three-dimensional (3-D) eye movement analysis. Acta Oto-Rhino-Laryngologica Belgica 1998;52:291-307.
4. Guidetti G, Trebbi M. The recurrences of paroxysmal positional vertigo. Audiological Medicine 2005;3:21-26.
5. Heidenreich KD, Kerber KA, Carender WJ, Basura GJ, Telian SA. Persistent positional nystagmus: a case of superior semicircular canal benign paroxysmal positional vertigo? Laryngoscope 2011;121:1818-1820.
6. Hornibrook J. Superior canal benign positional vertigo. New Zealand Medical Journal 2008;121:68-71.
7. Karatayli-Ozgursoy S, Stamper GC, Lundy LB, Zapala DA. Bilateral multicanal benign paroxysmal positional vertigo coexisting with a vestibular schwannoma: case report. Ear, Nose, & Throat Journal 2011;90:E10-15.
8. Meran A. [The neuro-otological findings of the multiple sclerosis (author's transl)]. Archives of Oto-Rhino-Laryngology 1977;214:351-359.
9. Obermann M, Bock E, Sabev N, et al. Long-term outcome of vertigo and dizziness associated disorders following treatment in specialized tertiary care: the Dizziness and Vertigo Registry (DiVeR) Study. J Neurol 2015;262:2083-2091.
10. Ogawa Y, Suzuki M, Otsuka K, et al. Positional and positioning down-beating nystagmus without central nervous system findings. Auris, Nasus, Larynx 2009;36:698-701.
11. Pula JH, Newman-Toker DE, Kattah JC. Multiple sclerosis as a cause of the acute vestibular syndrome. Journal of Neurology 2013;260:1649-1654.
12. Steddin S, Brandt T. Horizontal canal benign paroxysmal positioning vertigo (h-BPPV): Transition of canalolithiasis to cupulolithiasis. Annals of Neurology 1996;40:918-922.
13. Vanni S, Nazerian P, Casati C, et al. Can emergency physicians accurately and reliably assess acute vertigo in the emergency department? Emergency Medicine Australasia 2015;27:126-131.
14. Zapala DA. Down-beating nystagmus in anterior canal benign paroxysmal positional vertigo. Journal of the American Academy of Audiology 2008;19:257-266.

### Reason for exclusion: participants not central positional nystagmus subjects

1. Baloh RW, Yue Q, Jacobson KM, Honrubia V. Persistent direction-changing positional nystagmus: another variant of benign positional nystagmus? Neurology 1995;45:1297-1301.
2. Bisdorff AR, Debatisse D. Localizing signs in positional vertigo due to lateral canal cupulolithiasis. Neurology 2001;57:1085-1088.
3. Brookler KH. Possible central vestibular findings in a patient with a peripheral vestibular disorder. Ear, Nose, & Throat Journal 2004;83:520, 522.
4. Coats AC. Central electronystagmographic abnormalities. Archives of Otolaryngology 1970;92:43-53.
5. Herrmann R. [Is there central vestibular nystagmus in the bilateral absence of labyrinth function?]. Archiv fur Ohren-, Nasen- und Kehlkopfheilkunde, Vereinigt Mit Zeitschrift fur Hals-, Nasen- und Ohrenheilkunde 1963;181:149-153.
6. Kim CH, Shin JE, Song CI, Yoo MH, Park HJ. Vertical components of head-shaking nystagmus in vestibular neuritis, Meniere's disease and migrainous vertigo. Clinical otolaryngology : official journal of ENT-UK ; official journal of Netherlands Society for Oto-Rhino-Laryngology & Cervico-Facial Surgery 2014;39:261-265.
7. Kim H-J, Lee S-H, Park JH, Choi J-Y, Kim J-S. Isolated vestibular nuclear infarction: report of two cases and review of the literature. Journal of Neurology 2013;261:121-129.
8. Kim SH, Chung WK, Kim BG, Hwang CS, Kim MJ, Lee WS. Periodic alternating nystagmus of peripheral vestibular origin. Laryngoscope 2014;124:980-983.
9. Lechner C, Taylor RL, Todd C, et al. Causes and characteristics of horizontal positional nystagmus. Journal of Neurology 2014;261:1009-1017.
10. Lopez L, Bronstein AM, Gresty MA, Rudge P, du Boulay EP. Torsional nystagmus. A neuro-otological and MRI study of thirty-five cases. Brain 1992;115:1107-1124.
11. Mahringer A, Rambold HA. Caloric test and video-head-impulse: a study of vertigo/dizziness patients in a community hospital. European archives of oto-rhino-laryngology : official journal of the European Federation of Oto-Rhino-Laryngological Societies (EUFOS) : affiliated with the German Society for Oto-Rhino-Laryngology - Head and Neck Surgery 2014;271:463-472.
12. Okada M, Sato E, Nishihara E, Takagi T, Gyo K. Ten cases of positional down beat nystagmus without central nervous system findings. Equilibrium Research 2014;73:533-537.
13. van der Scheer-Horst ES, van Benthem PP, Bruintjes TD, van Leeuwen RB, van der Zaag-Loonen HJ. The efficacy of vestibular rehabilitation in patients with benign paroxysmal positional vertigo: a rapid review. Otolaryngology--head and neck surgery : official journal of American Academy of Otolaryngology-Head and Neck Surgery 2014;151:740-745.
14. Yang TH, Oh SY. Geotropic central paroxysmal positional nystagmus in a patient with human immunodeficiency virus encephalopathy. Journal of neuro-ophthalmology : the official journal of the North American Neuro-Ophthalmology Society 2014;34:159-161.

### Reason for exclusion: did not describe nystagmus

1. Bertholon P, Bronstein AM, Davies RA, Rudge P, Thilo KV. Positional down beating nystagmus in 50 patients: cerebellar disorders and possible anterior semicircular canalithiasis. Journal of Neurology, Neurosurgery & Psychiatry 2002;72:366-372.
2. Bhattacharjee S, Majumdar A, Jana A. Headache and central positioning vertigo in a middle aged female-a case of solitary cerebellar tuberculoma involving left cerebellar hemisphere. Turk Noroloji Dergisi 2012;18:39-42.
3. Cawthorne T, Hinchcliffe R. Positional nystagmus of the central type as evidence of subtentorial metastases. Brain 1961;84:415-426.
4. Chang MB, Bath AP, Rutka JA. Are all atypical positional nystagmus patterns reflective of central pathology? Journal of Otolaryngology 2001;30:280-282.
5. Choi JY, Park YM, Woo YS, Kim SU, Jung JM, Kwon DY. Perverted head-shaking and positional downbeat nystagmus in pregabalin intoxication. Journal of the Neurological Sciences 2014;337:243-244.
6. Fisher A, Gresty M, Chambers B, Rudge P. Primary position upbeating nystagmus. A variety of central positional nystagmus. Brain 1983;106:949-964.
7. Gerhardt HJ, Meyer ED, Werbs M, Biedermann F. Early diagnosis of acoustic neurilemmomas. [German]
8. Zur Fruhdiagnose Des Akustikusneurilemmoms. HNO-Praxis 1985;10:91-95.
9. Jannetta PJ, Moller MB, Moller AR. Disabling positional vertigo. New England Journal of Medicine 1984;310:1700-1705.
10. Kumar A, Patni AH, Charbel F. The Chiari I malformation and the neurotologist. Otology & Neurotology 2002;23:727-735.
11. Petrova D. Nystagmus reactions among patients with asymptomatic cerebrovascular diseases: results and analysis of investigation of 228 persons. International Tinnitus Journal 2005;11:177-180.
12. Rosemergy I, Mossman S. Brainstem lesions presenting with nausea and vomiting. New Zealand Medical Journal 1254;120.
13. Shimizu N, Mizuno M. Oculomotor characteristics of parkinsonism in comparison with those of cerebellar ataxia. Journal of Neural Transmission Supplementum 1983;19:233-242.
14. Smouha EE, Roussos C. Atypical forms of paroxysmal positional nystagmus. Ear, Nose, & Throat Journal 1995;74:649-656.
15. Sweeney PJ, Hahn JF, McHenry MC, Mitsumoto H. Mucormycosis presenting as positional nystagmus and hydrocephalus. Case report. Journal of Neurosurgery 1980;52:270-272.
16. Thomsen J, Tos M. Vestibular and other oto-neurological findings in acoustic neuromas. Acta Oto-Laryngologica 1984;98:64-67.
17. Urban PP, Horwath K, Wellach I, Pohlmann C, Bruning R. [Central positional vertigo due to cerebellar nodular infarction]. Nervenarzt 2009;80:948-952.
18. Wang CP, Young YH. Audiovestibular symptoms in a case of primary intraocular lymphoma with brainstem metastasis - A case report. Tzu Chi Medical Journal 2002;14:109-112.
19. Yu-Wai-Man P, Gorman G, Bateman DE, Leigh RJ, Chinnery PF. Vertigo and vestibular abnormalities in spinocerebellar ataxia type 6. Journal of Neurology 2009;256:78-82.

### Reason for exclusion: positional tests not performed

1. Alpini D, Caputo D, Pugnetti L, Giuliano DA, Cesarani A. Vertigo and multiple sclerosis: Aspects of differential diagnosis. Neurological Sciences 2001;22:S84-S87.
2. Dix MR. Clinical observations upon the vestibular responses in certain disorders of the central nervous system. Advances in Oto-Rhino-Laryngology 1970;17:118-128.
3. Harrison MS, Ozsahinoglu C. Positional vertigo. Archives of Otolaryngology 1975;101:675-678.
4. Jen JC, Yue Q, Karrim J, Nelson SF, Baloh RW. Spinocerebellar ataxia type 6 with positional vertigo and acetazolamide responsive episodic ataxia. Journal of Neurology Neurosurgery and Psychiatry 1998;65:565-568.
5. Kishi M, Sakakibara R, Yoshida T, et al. Visual suppression is impaired in spinocerebellar ataxia type 6 but preserved in benign paroxysmal positional vertigo. Diagnostics 2012;2:52-56.
6. Tilikete C, Milea D, Pierrot-Deseilligny C. Upbeat nystagmus from a demyelinating lesion in the caudal pons. Journal of Neuro-Ophthalmology 2008;28:202-206.
7. Weber PC, Cass SP. Neurotologic manifestations of Chiari 1 malformation. Otolaryngology - Head & Neck Surgery 1993;109:853-860.

### Reason for exclusion: unable to obtain full text

1. Kattah JC, Kolsky MP, Luessenhop AJ. Positional vertigo and the cerebellar vermis. Neurology 1984;34:527-529.
2. Lechner C, Taylor RL, Todd C, Welgampola MS. Characteristics of horizontal positional nystagmus. Journal of the Neurological Sciences 2013;333:e651-e652.
3. Vanni S, Casati C, Nazerian P, et al. Diagnosis of stroke in the acute vertiginous patient: A bedside three steps tool in the emergency department. European Heart Journal 2012;33:232.
